# Behavioral and Perceptual Differences between Sexes in Dogs: An Overview

**DOI:** 10.3390/ani8090151

**Published:** 2018-08-23

**Authors:** Anna Scandurra, Alessandra Alterisio, Anna Di Cosmo, Biagio D’Aniello

**Affiliations:** Department of Biology, University of Naples “Federico II”, 80126 Naples, Italy; annascan@hotmail.it (A.S.); alessandra.alterisio@unina.it (A.A.); dicosmo@unina.it (A.D.C.)

**Keywords:** dog behavior, aggressiveness, boldness, navigation strategy, distractibility, lateralization, sociability, excitability, olfactory skill

## Abstract

**Simple Summary:**

We explore the differences in male and female dogs regarding personality traits as well as cognitive and perceptual processes. Our aim was to explore whether the differences in male and female dogs were affected by the domestication process. The results show that dogs are largely in line with the life-history theories, reflecting the sex differences described in wild animals.

**Abstract:**

In this paper, we review the scientific reports of sex-related differences in dogs as compared to the outcomes described for wild animals. Our aim was to explore whether the differences in male and female dogs were affected by the domestication process, in which artificial selection is the main driver. For this purpose, we used information regarding personality traits, cognitive processes, and perception, for which there is a wide theoretical framework in behavioral ecology. Aggressiveness and boldness, described as a behavioral syndrome, were reported as being higher in males than females. Females also seemed more inclined to interspecific social interactions with humans in tasks that require cooperative skills, whereas males appeared more inclined to social play, thus implying different levels of social engagement between the sexes, depending on the context. Studies on cognitive processes underlined a greater flexibility in resorting to a particular navigation strategy in males. Most lateralization studies seem to support the view that males are preferentially left-handed and females are preferentially right-handed. Reports on visual focusing coherently rank females as superior in focusing on single social and physical stimuli. Only male dogs are able to discriminate kin; however, the timing of the olfactory recording in sexes is related to the stimulus relevance. Dogs are largely in line with life-history theories, which indicate that sex differences in dogs are mainly rooted in their biological and evolutionary heritage, remaining unchanged despite artificial selection. In contrast, the higher intraspecific sociability in wild male animals was not replicated in dogs.

## 1. Introduction

In behavioral ecology, there has been increased interest in studies on individual behavioral differences in animals. Examples include studies of behavioral phenotypes, temperaments, or personalities in both vertebrate and non-vertebrate species (refer to [1] for a review). These studies have underlined several inter-individual differences in different traits, such as aggressiveness [2,3], activity levels [4,5], sociability [3], and boldness [6]. Individual competition may favor the expression of traits that improve reproductive fitness, although these specific adaptations are often costly in terms of energy and survival at both the morpho-physiological and behavioral levels [7,8,9].

Sex status is a biological trait that affects the determination of an individual’s behavioral responses to physical and social environmental challenges, thus biasing the behavior of the sexes. The reproductive success of males and females depends on different factors. In the majority of studied species, females are more limited by the production and care of offspring, and their fitness is not enhanced by mating with multiple males, whereas males fitness is directly proportional to the number of females inseminated [7,10,11,12,13,14]. Moreover, specific traits may be actively selected and maintained by sexual selection. For example, differences in personality traits between males and females animals have been linked to sexual selection as an effect of intra-sexual competition and mate choice in both humans and non-human animals [15].

In humans, cognitive processes such as visual-spatial perceptions and verbal and mathematical approaches follow different brain processing in men and women, although disputes remain with respect to this observation [16,17]. Ethological studies also underline many behavioral sex differences in other animals [18]. Prominent observations related to reproductive behaviors, such as parental care, mating strategies, and courtship displays, are almost exclusively expressed by only one of the sexes. These traits have been tagged as real “sexual dimorphism” [19] or “qualitative differences” [18]. However, differences in behaviors not exclusive to reproduction are less obvious and may differ in magnitude between the sexes. Odor detection and stress responses, for example, fall in this category and are simply considered “sex differences” [19] or “quantitative differences” [18]. In some cases, both sexes appear to exhibit the same behavior; however, the underlying neural substrate differs between them such that, under particular conditions, one sex might display a different behavior (sex convergence and divergence, [19]). For example, Lighthall et al. [20] reported there were no significant sex differences in a human decision-making task; however, under the influence of a cold pressor stress, men showed a faster reward-related decision-making speed than females, thus indicating a clear sexual divergence in behavior. This effect was attributed to differential brain functions in the dorsal striatum and anterior insula, with an increased activation in men compared to women after the stress event. Finally, there may also be “population differences” in behavior, which indicates that the frequency of display varies between the sexes, although the pattern is consistent [18]. For example, in most social mammals, males tend to disperse more than females [21].

The dog (*Canis lupus familiaris*) has evolved into a synanthropic species via a very long domestication process over the ages, which involved both natural and artificial selection. Co-evolution with humans has shaped the dog’s cognitive processes accordingly, favoring behaviors that aim to optimize their adaptation to various anthropogenic environments. Dogs implement appropriate behavioral strategies in response to communicative cues from humans through different sensory channels. They are responsive to both verbal and non-verbal vocal sounds [22] and can recognize up to several hundred words [23,24], with specific neural mechanisms that analyze and integrate word meaning and intonation [25]. They have also evolved an acute sensitivity to human gestures [26,27,28,29,30]. Moreover, it has recently been demonstrated that dogs are able to perceive human emotions via chemosignals, which suggests a type of olfactory communication [31,32].

How dogs acquired these skills remains a subject of debate. The “domestication hypothesis” emphasizes genetic predispositions that may have enabled dogs to develop communicative skills attuned to humans [33,34,35,36], whereas the “two-stage hypothesis” leans more on ontogenetic aspects [37,38,39], implying that dogs may have learned from humans during their ontogenesis, thus shaping their behavioral responses [40,41] and improving their social communicative skills [30,42]. These two theories have been integrated into the “synergistic hypothesis,” which suggests that sensitivity to human gestural cues may have emerged at both the evolutionary and developmental levels [43], although individual contributions of genetic and ontogenetic inputs have yet to be determined. The switch from natural (and sexual) selection to artificial selection may have imposed several deviations from what might be regarded as the natural situation. Co-habitation with humans may have directly diminished the selective pressure in dogs for essential survival traits [44]. For example, although dogs are able to utilize intraspecific observational learning [45,46], they have been shown to be less skillful than wolves in this behavior [47]. As a side effect of artificial selection, the differences between males and females, which are maintained in nature through natural (and sexual) selection, could have changed during and after domestication. Furthermore, living often in close proximity with and depending by humans could have make less necessary to maintain sex-specific traits. In such a context, the dog may be an interesting model to investigate the effect of human directed effects on the roles of males and females and their interactions. Thus, the aim of this review is to describe whether the sex-specific differences identified in wild animals were affected by living in the anthropogenic niche in dogs, which include the domestication process and the ontogenetic acquisitions.

Papers on sex differences in dogs were first selected in our literature database, which is monthly updated regarding studies on dog behavior, periodically checking the journals included in the “behavioral science,” “zoology” and “multidisciplinary sciences” categories. We subsequently enlarged our sample by specifically searching in the main online academic databases for “dogs sex differences” or “sex differences” coupled with keywords related to the specific personality trait, behavior or perceptive channel (e.g., aggress; behav phenotype, behav syndrome, behav trait, bold, domestication, fear, neophobia, olfactory, perception, personality, predator, selective strategy, sociability, temperament and visual focusing). Papers of interest were selected (e.g., physiological differences were excluded), and duplicates were eliminated. A further search was performed by reviewing the references in the selected papers, which revealed other missing studies (Table 1).

The studies have been compared and summarized according to our experience and framed in the theories of behavioral ecology. Several specific sex-related differences in dogs regarding personality traits (i.e., excitability and distractibility) and cognitive processes (i.e., smartness) for which naturalistic frameworks do not yet exist have been excluded.

## 2. Personality Traits

### 2.1. Aggressiveness

Aggressiveness is a hostile behavior (e.g., threatening gestures or real attacks) that may inflict physical or emotional harm to one or more different targets and is performed with the intention to modify the behavior of a recipient. Intraspecific aggression is directed toward members of the same species, whereas interspecific aggressiveness is directed toward members of different species. Bouts of aggression may also be elicited following a threatening event not related to another living being. Displays of aggression are linked to the instinct of preservation and are well documented in predation scenarios, often evoking an aggressive defensive response (fear-induced) in the prey species. Aggression may also be aimed at defending a territory for monopolizing resources (e.g., food and mating partners) or achieving and maintaining a higher social status. Thus, from a behavioral ecology perspective, aggression is a tool to achieve a competitive advantage; however, it is a behavior that is energetically expensive, time-consuming and potentially dangerous [7,12]. The energy employed in the expression of the aggressive behavior is no longer available for other functions, such as pregnancy and caring for offspring. For males, the cost of the aggressiveness to defend a territory and obtain access to females is balanced by ameliorative reproductive success, whereas females have fewer direct advantages, considering that the energy invested in the expression of aggressive displays is detracted by the functions related to the sex-specific behaviors linked to reproduction [48]. Thus, it is expected that females would express fewer aggressive behaviors than males in several contexts. In many species, including humans, a higher incidence of aggressive behavior has been reported in males and is well documented [49,50]. However, in some species (e.g., pigs), males and females show the same level of aggressive behaviors [51] and in some cases, a sex-reversed trait, with more aggressive females, has also been identified. For example, as an effect of the particular social structure in which females are dominant, female spotted hyenas (*Crocuta crocuta*) appear to be consistently more aggressive than males [52]. Considering that dogs do not belong to a species with a sex-reversed role, more aggressive behavior is expected in males unless the domestication process has affected sex differences related to aggression.

Dogs often live in close relationship to human families, in which aggression would be an unwanted behavioral trait. It has been established that aggression is one of the most complex canine behaviors to define in terms of context, intensity, and target [53]. Intraspecific aggression in dogs has long been acknowledged [54] and represents a major behavioral problem, together with interspecific aggression toward both unfamiliar and familiar humans [53]. Furthermore, aggressiveness is required in specific working dogs, such as military and guard dogs, although they always work under human supervision. In dogs, displays of aggression generally involve barking, growling, and biting, which are often exhibited in an escalating sequence and are accompanied or preceded by threatening or defensive postures, such as standing-over, staring, lunging, ears being pulled back, and the tail held down. The tendency for aggression seems to be a result of both environmental and genetic factors [55,56] and may be modulated by life experiences. Dogs that are appropriately socialized as puppies are less likely to exhibit aggression [57,58]. One of the oldest studies addressing sex differences in aggressive behavior in dogs was based on direct interviews with the owners [59]. Several hundred cases involving aggression in dogs kept as companion animals were considered. The study aimed to delineate motivations that elicit aggressive responses and examined differences in the reactivity of males and females on a case by case basis. The results indicated that intraspecific aggression is the major motivations influenced by sex, with males expressing higher levels than females. In intraspecific aggression, females appeared to be aggressive predominantly toward other females. Aggression incidences have been reported to be higher in males than in females in many other studies [60,61,62,63,64,65,66].

A more recent report, aimed to investigate the effects of early maternal and litter factors on different behavioral traits measured in adult German Shepherds, showed a principal component (i.e., aggression) with a loading of 0.62. Females scored lower than males on aggression [67]. Displays of aggression were the results of an environmentally threatening event not related to another living being; thus, this study could not provide information regarding intra- or interspecific aggressiveness. Furthermore, a study on the personalities of Labrador Retrievers demonstrated a lower tendency of females, compared to males, in engaging aggressive behaviors toward the owner, whereas no differences between sexes were found for stranger-directed and intraspecific aggressions [68]. In the same study, male German Shepherd dogs were classified as generally more aggressive than females. Intraspecific aggressiveness was not specifically tested in the latter study, a matter addressed by Rooney and Bradshaw [69], which demonstrated that English Springer Spaniel, Labrador Retriever, cross-breeds, and Border Collie males were more aggressive toward other male dogs than conspecific females. In another study with 20 different breeds, investigators found that male dogs generally showed more aggression toward both other dogs and human strangers [70].

The effect of sex hormones in regulating aggressive behaviors is somewhat more complex. Several studies have reported that castration reduces intraspecific aggressive behaviors between males [59,66,71,72,73,74] as well as interspecific aggressive behaviors toward humans [75]. These outcomes concur with data that indicate dominance-related aggressiveness correlates with the levels of androgens in pet dogs [50]. However, in other studies, observations that castration reduced intraspecific [71,76,77,78,79,80,81] or interspecific aggression in male dogs [82] could not be confirmed. Rather, increased aggression in castrated dogs was also identified in some cases [76,83,84,85]. Studies on female dogs appear to be more coherent, as a majority of outcomes report a higher level of aggressive behaviors in spayed females [59,66,75,86,87,88]. However, a recent study failed to replicate these data and indicated a contradictory lower incidence of aggression toward people (i.e., both familiars and strangers) in spayed female dogs [80]. 

### 2.2. Boldness and Courage

In laymen’s terms, words such as fearlessness, courage, bravery, dauntlessness, intrepidity, or boldness are often considered synonyms that indicate a particular mindset that equips one to face difficulty or danger. In human psychology, each of these terms has a distinct meaning. Courage, for example, as opposed to fearlessness, is identified as a behavioral approach to a task despite the feeling of fear [89], in the sense that a courageous individual could complete the same action as a fearless individual, despite experiencing fear [90]. Personality studies consider boldness a “super-trait” identifying higher-order personality traits in humans and other animals [91,92], belonging to one end of the shy-bold axis in dogs [93,94]. According to the risk-reward hypothesis [95], boldness makes individuals more proactive and explorative, which enables greater potential to gather resources; at the same time, it enforces more risks. Shyer individuals take fewer risks; however, they lose opportunities for foraging and mating, thereby reducing fitness [96,97,98]. In the species in which the mating success of males depends on the time spent on the courtship, searching for females, and competing for access to partners, higher boldness is required to face the risk of being detected and caught by predators. In contrast, females may choose mates according to boldness, thus advantaging bolder males through sexual selection [15]. In this scenario, it is expected that males will be bolder than females. Studies across vertebrates (fish: [99,100,101,102]; reptiles [103]; birds: [104,105]; mammals [106,107]) have coherently demonstrated a higher boldness in males compared to females. However, the direction of the sex difference for this personality trait is likely to depend upon ecological factors, as in the case of the hyena females that turned out to be bolder than the males [108]. In invertebrates, females appear systematically bolder than males [109,110].

It has been observed in many species that boldness correlates with aggressiveness: individuals more likely to take risks by engaging in intraspecific aggressive fights also appear to risk more when confronted with environmental hazards, such as predators [108,111,112,113]. This recurrent correlation has enabled scholars to individuate a specific aggression–boldness syndrome [1]. Thus, considering that most of the researches report a higher level of aggressiveness, it is expected that male dogs will exhibit greater boldness, which proved to be the case in the research on the matter. In dogs, boldness is described as an individual characteristic providing less aversion to risk or novelty that enables the subject to actively seek out and engage in social interactions (at both cooperative and competitive levels), as well as toward non-social objects or events [93,94,114,115,116,117,118,119]. One of the main components to consider when defining boldness seems to be the level of neophobia [120], although it has not been considered as an indicator of some investigations [121].

We have attempted to consolidate all studies related to sex differences that specifically discuss boldness, including studies concerning fear responses and courage. One of the first studies conducted in relation to sex difference–related fear responses was an investigation of several components of behavior used to select Alsatian dogs (e.g., German shepherds) for the Swedish Army [122]. Based on the trainers’ evaluations, the authors reported that there were no differences between males and females in courage (measured as a response to an approaching man-shaped figure) and responses to sudden disturbances. However, it was noted as a sub-classification of the latter response that females were more susceptible than males to gunfire. In the following study aimed at determining the factors that affect the suitability of subjects as guides for blind people, female guide dogs (mostly Labrador and Golden Retrievers) were rejected more often than males because of problems related to fearfulness [123,124]. The latter studies were based on the trainer’s scoring, in which the authors attempted to correlate behavioral and genetic traits with influencing environmental factors. Investigations based on trainer assessments are often considered inconsistent [125], inasmuch as scores vary greatly in consistency between trainers [124]. However, these findings were confirmed in a follow-up study based on experimental designs aimed at evaluating the quality of puppy walking that guide dogs experienced, correlated with measurements of exploration and activity [126]. The follow-up study was conducted on Labrador Retrievers, German Shepherds, Boxers, Kelpies, and F1 crosses, with dogs tested at six and 12 months of age (during the puppy walking time) and again when they were returned to the Guide Dog Center for training (final testing). The tests measured different behaviors related to the general activity of the dogs and the willingness to respond to commands. Goddard and Beilharz [126] determined that in unfamiliar, crowded, and noisy places both olfactory exploration and neophobia were increased. In these circumstances, females showed higher levels of olfactory exploration than males, thus indicating a heightened fear response. In a study by Wilsson and Sundgren [127] on different behavioral characteristics in Labrador Retrievers and German Shepherds, males scored higher than females in courage in both breeds, as evaluated by seven test situations. Results from further studies regarding boldness and fearlessness as behavioral traits fall along the same lines. One study investigated the correlation between the personality and performance of Belgian Tervurens and German Shepherds in working dog trials [94]. The dogs were subjected to a variety of different tests, and the sexes were compared using a boldness score, which was extracted by a factor analysis of different behavioral outcomes. Males scored higher than females. Similar results were obtained in a study that aimed to examine the genetic covariation of behavioral traits in German Shepherds, in which males appeared bolder than females [128], as indicated by dog mentality assessment tests [129].

In another study, using 14,004 questionnaires on different breeds directed to owners in Germany, Kubinyi et al. [130] applied a principal component analysis to 24 items, obtaining the boldness factor described by the traits reserved, aloof, and fearful with scores of up to 0.7. The following analyses demonstrated that boldness was age-dependent, with younger male dogs (younger than two years) scoring higher on the boldness factor scale than older dogs or female dogs. Overall, intact males were the boldest group, whereas spayed females were the least bold. Similar results obtained from personality surveys circulated among Australian dog owners [131] also confirmed the negative effect of neutering on boldness in both sexes. A principal component analysis produced “boldness” as a factor in which social traits scored higher positive loadings, whereas avoidance and other fear-related behaviors showed higher negative loadings. In a recent study [70], breed and grouping effects (working/non-working) on everyday behavior, in a sample of 20 different breeds of Swedish dogs, showed that male dogs exhibited fewer conspecific-directed (fearful response to unfamiliar dogs) and stranger-directed (fearful response to unfamiliar person) fears compared to female dogs, whereas no significant sex difference toward the owner was observed.

### 2.3. Sociability

Many species live in complex social structures in which affiliative interactions prevail against anti-social behaviors, such as aggression and territoriality. In humans, sociability is defined as an attitude of taking into account other individuals to achieve a goal. In the animal studies, Reale et al. [96] provided a terminology for the sociability to be used as a working tool for ecological studies of temperament. According to the authors, the “sociability is an individual’s reaction to the presence or absence of conspecifics (excluding aggressive behavior). Sociable individuals seek the presence of conspecifics, while unsociable individuals avoid conspecifics.” 

In the realm of behavioral ecology, it is proposed that the social behavior of males and females is differentially targeted by selective pressures. Males are principally devoted to access to females, whereas females privilege other resources [132,133]. In this context, males should tend to be more aggressive than females in social behavior because the socioecological theory predicts that social contacts in males increase reproductive success, thus enabling the animal to reach a high rank in the hierarchy or establish alliances [134,135]. In many species, such as primates [136,137] and dolphins [138], males establish alliances, whereas female dolphins have been observed to form more dynamic social bonds [139,140]. In some primate species, males appear to develop greater social behavior than females very early in life: male infant chimpanzees show more social interaction than females, and they also interact with more adult males than females [141]. In the same way, human boys have been reported to be more social, playing in larger groups than girls [142,143]. In contrast, in a study on intraspecific sociability, male dogs appeared to be less sociable than females, with a pronounced effect in dogs belonging to the 4–8 years age group [131]. In the latter study, sociability was extracted by four traits (i.e., friendly, quarreling, bullying, and kindness) that received high loading in a principal component analysis.

Although studies of animal intraspecific sociability in wild and captive animals have been abundant, interspecific sociability has barely been investigated, which may be a result of the difficulty of studying cooperating species. In this context, considering their long cooperative story with humans, dogs are very appropriate models. A recent study has suggested that canine sociability may be the result of the canine homologous Williams-Beuren syndrome [144], a genetic disorder that in humans causes hyper-sociability, among other symptoms [145]. Despite the overall high sociability in dogs, there are indications that females may be more likely to interactions with humans. One of the first reports showing sex differences in sociability was performed in 20 intact pet dogs of different pure and mixed breeds [146]. The dogs’ reactions to an unfamiliar person were assessed by their responses to male and female human-reaction tests. The results showed that female dogs were friendlier and would make physical contact with a stranger. 

Support for the hypothesis that female dogs are more social in interspecific interactions was obtained in the context of results from tests that assessed decision-making mechanisms, such as the impossible task paradigm. The impossible task paradigm [147] is similar to the problem-solving paradigm: the subject initially learns to solve an easy task, which in the next phase of the test becomes impossible to solve, thus raising an expectancy violation that forces the subject to pursue the objective alone or ask for help from human counterparts [148]. This paradigm has been very useful to investigate canine social interactions with known people and strangers [40,41,42]. Researchers have applied this paradigm to investigate the responses of a Beagle population living in a kennel under standard conditions [149]. The participants did not have a precise reference figure, as the researcher was the only human reference in the test. The results showed that females outperformed males when they encountered the impossible phase of the task, with higher social interactions with the experimenter, including alternating interactions between the apparatus and the experimenter. Although the study included only one breed, a large sample size (*n* = 498) was tested, indicating that the tendency for higher sociability observed in females may be genetically encoded [149]. Similar results were obtained in our work with the impossible task paradigm applied to different breeds [150]. Our experimental setting provided an option of two human partners, the owner and a stranger, who did not touch the container or the food (refer to [40,41] for details). Re-analyzing our database to filter for the sex of the dogs, we determined that, as previously reported [149], females were more willing to interact with the stranger than males, whereas no sex differences were identified for the owner. Further support for a higher tendency among female dogs for socialization with humans was obtained from a study on German Shepherds that aimed to evaluate the correlation of early maternal and litter traits with different behavioral traits measured in adult dogs at the Swedish Armed Forces [67]. Behavioral traits were extracted from a temperamental test used by the armed forces to select suitable work dogs. The Swedish Armed Forces and the researchers [127] used a modified version of the Dog Mentality Assessment test, including 12 standardized sub-tests. A principal component analysis showed that female German Shepherds scored significantly higher than males in social engagement. 

Despite this body of evidence highlighting that female dogs engage more in social interaction with humans, other data seem to be contradictory. Using a questionnaire distributed to dog owners, Asp et al. [70] investigated 20 breeds registered at the Swedish Kennel Club, limiting the bias from subjective descriptions of a dog by using a large number of independent discrete responses (refer to [151]). In this study, male dogs were found to be more interested in human-directed play. These results mirrored the findings obtained in Dog Mentality Assessment standardized tests on German Shepherds used by the Swedish Working Dog Association, in which males scored higher in social play than females [128].

## 3. Cognitive Processes

### 3.1. Spatial Cognition

Spatial cognition is the internal understanding and recollection of space [152] and concerns the study of our awareness of objects and events in the world [153]. Spatial navigation is a process that enables animals to know their surroundings and identify the optimal path to their targets using multiple resources such as path integration, magnetic cues, and different landmarks [154]. This process involves memorizing specific landmarks, positions, and locations to create a cognitive map that enables one to orient and navigate oneself through the surrounding environment.

In mammals, males typically show greater prowess in spatial navigation tasks, likely because of a relevant function in reproduction [155,156]. The males’ advantage in solving spatial navigation tasks has been linked to the competition for mating, which often requires a larger territory [157,158], whereas the females’ major involvement in reproduction may have favored a superior spatial sense in more restricted areas [158,159]. Males utilize an allocentric navigation strategy based on the relative positions of environmental landmarks, whereas females rely more on an egocentric navigation strategy, predominantly referring to their motor responses [159,160,161,162].

Using a wide range of spatial skills, dogs can solve different spatial tasks, including both egocentric and allocentric signals depending on the task [163,164]. They can integrate spatial signals during locomotion, continuously updating information on the distance from and direction to a particular object (path integration; [164]). Based on studies regarding mammals, it is expected that the use of navigation strategies differs in male and female dogs. In a study that investigated dogs’ flexibility in the acquisition of spatial information through social learning [165], the dogs were tested in the “Do as I Do” paradigm [166] that required the dogs to reproduce actions demonstrated by humans. First, it was determined whether the dogs preferred an egocentric or allocentric strategy for recalling the demonstration of the owner. Once an allocentric strategy was confirmed in this context, dogs were forced to switch to an egocentric strategy by rotating the targets by 90% and withholding a reward until it touched the correct (egocentric) target. It was shown that male dogs switched from the preferred (allocentric) to non-preferred (egocentric) strategy in fewer trials than females. Another study tested the navigation skills of dogs in an indoor T-maze paradigm [167]. The dogs’ ability to learn the correct exit path from the maze was initially first tested, and a recall memory test was performed after two weeks to assess whether they retained the information acquired in the learning task. Finally, the dogs were tested in a reversal-learning task that aimed to evaluate the dogs’ ability to modify previously acquired information regarding the correct exit path. The results showed that intact females had a better performance in the learning task than ovariectomized females and intact males. In a third study by the same group [168] dogs’ spatial skills were tested in an indoor plus-maze. After a learning phase to acquaint the dogs with the location of food in the maze, their preference toward egocentric or allocentric information was assessed. The dogs subsequently underwent a reversal-learning phase to force them to change their preferred navigation strategy. Sex-related differences were absent from the strategy preference in such a context. However, ovariectomized females were significantly more likely to prefer an egocentric strategy. An interesting result in the plus-maze study was that the probability of successfully resorting to the non-preferred strategy increases with age in females, whereas it decreases in males [168].

### 3.2. Lateralization

Lateralization has been extensively investigated as a physical measure of the brain’s asymmetry [169,170,171] and is manifested as a bias in performing motor or sensory tasks based on the dominant hemisphere [172]. It has been established that the left hemisphere of the brain controls the expression of behavioral patterns in non-stressful situations, whereas the right hemisphere controls behaviors in unexpected or dangerous situations that require fight and flight responses [173]. In some vertebrates, it has been reported that aggressive responses toward conspecifics are performed predominantly from their left side [174,175,176].

In dogs, lateralization has been identified in different functions. Studies have shown that domestic dogs display a left gaze bias when viewing human faces [177] and that the emotional valence of facial expressions affected this behavior [178]. Similarly, dogs show a lateralization of tail-wagging [179] and olfactory [31] and acoustic processing [180] following negative and positive stimuli, thus providing support for hemispheric specialization.

A paw preference test paradigm is a common tool adopted for the study of motor function lateralization. Sex differences in paw preferences have been reported in cats, with females showing greater use of their right paw, whereas males preferred their left paw [181,182,183]. Similar outcomes were also reported in primates [184] and horses [185]. In humans, a meta-analysis of studies on handedness indicated more left-handedness in males [186]. This pattern of different lateralization in males and females is challenging to interpret, particularly in light of the theory that bilateral symmetry is an evolutionary adaptation [187]. Most environmental actions could be better solved by having the opportunity to use both paws indifferently, without the need to change the position of the body to enable the use of a dominant limb. Thus, it should be expected that animals would not show paw preferences, irrespective of sex. However, considering the previous findings in domesticated species and primates it is expected that there is a higher number of left-pawed male dogs than females.

One of the first studies that addressed paw preferences in dogs demonstrated that more than 50% were right biased, approximatively 18% were left-biased, and 25% were ambidextrous [188]. The subjects were blindfolded with adhesive plaster and subsequently allowed to attempt to remove it from the eyes using their preferred paw. In another study, dogs were tested in three different tasks [189]. The first task was an arbitrary action in which the participants were instructed to give a paw after sitting, the second task involved an action directed toward a flannel blanket that the dogs had to remove from over their heads, and the third task was a food retrieval task from a metal can. Interestingly, males and females showed contrasting paw preferences, in which females preferred their right paw, whereas males were biased to their left paw. These results were confirmed in a follow-up study in which dogs were required to remove a piece of adhesive paper from the snout [190]. In a more recent study, paw preferences were tested using a Kong toy, a hollow conical-shaped rubber toy stuffed with food [191]. However, in this case, the results were contradictory to previous studies, as significantly more male dogs were classified as right-pawed, whereas females were shown to be ambidextrous. Despite the assumption that the paw used to stabilize the Kong was the dominant paw, the researchers claimed that the participants used their non-preferred paw to stabilize the toy, which mirrors the results obtained by similar work on lateral limb use in humans [192]. Although this interpretation of the results is in line with the previous analogous human studies, other research groups have not yet been able to replicate these data. Branson and Rogers [193] and Schneider et al. [194] also investigated paw preferences using the same Kong paradigm of Wells et al. [191]; however, they did not substantiate differences in paw preference between males and females. Poyser et al. [195] also tested dogs in different tasks that replicated the paradigms of Wells [189] and Quaranta et al. [190] and did not identify a significant association between paw preference and sex. In line with previous studies, Poyser et al. [195] underlined a tendency of males to use the left paw in the first trial with a significantly shorter latency of usage. However, this effect was less pronounced with repeated presentations of the test and was not recorded in females, thus prompting the interpretation that behavioral lateralization was labile in dogs and might be influenced by hemispheric effects only responding to novel stimuli.

In an attempt to interpret the inconsistency in the literature, Tomkins et al. [196] considered that different tests could produce different results regarding lateralization. For example, the Kong test focused on food retrieval and tape-removal with the aim to relieve discomfort may have posed different challenges to the participants and thereby influenced the effects [172]. Moreover, studies have suggested that task complexity may influence the results of lateralization paradigms [197,198]. 

## 4. Perception

### 4.1. Visual Focusing

Visual focusing is an animal’s ability to concentrate and distinguish significant perceptual cues in the immediate physical environment at any given time. In humans, a study aimed to reveal sex differences in exploratory eye movements measured the exploratory eye movements of normal subjects (39 male and 39 female) using an eye-mark recorder. A wide set of the picture was projected onto a screen (e.g., open circle, happy face, different animals, the sun, an airplane, five trees, a house, two mountains, and a river). The results revealed that the mean gazing time of adult women looking at pictures was consistently longer than that of men, while the environmental scanning length of adult men was higher [199]. This finding may be an effect of the greater need for vigilant behavior in men [200] as well as in other male animals [201,202]. Based on the results obtained in humans, it is expected that female dogs will have a greater tendency to focus on a single target.

In the context of social cognition, a study on shepherds and Molossoid dogs showed that when an unfamiliar person approached, females displayed more referential gazing and gaze alterations between the owner and the stranger [203]. Similarly, Mongillo et al. [204] reported that females looked more at their owners in a room where the owner was talking to a stranger. Although castrated males were not included in the cited study, intact females showed more gazing behavior than spayed females, thus also underlining a potential effect of female sex hormones in gazing behavior. Females also seem to be responsive to intranasal oxytocin increasing the gazing behavior toward the owner [205,206]; however, sex differences with regards to oxytocin have not been reported in other studies (e.g., [207]). Female dogs also seem to rely more on visual signals than males in their behavioral regulation. A study that aimed to assess the preferred communicative channel between verbal and gestural messages underlined a preference for visual cues in dogs [26]. A group of Labrador and Golden Retrievers, after acclimatization to four common actions (i.e., sit, down, stay, and come) using bimodal gestural and verbal messages, were subjected to three different conditions. The dogs were required to perform the actions using verbal commands only, gestural commands only, and then in conflicting bimodal condition in which the gestural and verbal messages were directed toward opposite actions. The results showed that in the absence of visual signals, females made more mistakes than males, which indicates a greater dependence of female dogs on visual cues.

Physical cognition is concerned with the understanding of and interaction with the physical world and the different objects in it. The studies in this domain typically focus on an animal’s problem-solving skills with tools and processing the considerable complexity of their environment. In a study by Müller et al. [208], male and female dogs were tested in an object permanence task in the expectancy-violation paradigm. Unexpected and expected events were presented to the dogs: a ball disappearing behind a screen and another ball of different (unexpected) or the same (expected) size reappearing on the other side. Females reported longer gazing times than males in the unexpected than in the expected conditions, thus demonstrating that females respond better to object permanence violation. The effect was independent of sterilization status in both sexes. In another study, dogs and different ape species were compared in their physical cognitive abilities using a transposition task under the same expectancy-violation paradigm [209]. No significant differences were identified between males and females; however, females showed a trend (*p* < 0.069) toward better performance in the object permanence task. In contrast to social cognition [204], sex differences identified in physical cognition appeared to be independent of sex hormones, as sterilization had no effect on performance [208]. These differences may be related to different information-processing strategies between the sexes that are acquired as a brain organizational effect in early life [208].

### 4.2. Olfactory Skills

Kin discrimination in animals is closely linked to various social and genetic benefits, including preferential treatment of kin (nepotism) in parental care or cooperative behavior, which facilitates a functional social structure [210]. Furthermore, kin discrimination avoids inbreeding that reduces individual fitness resulting from reproduction among relatives, a well-established phenomenon in both natural and experimental populations [211,212], although kin are preferred mate partners in some species (e.g., Cichlid fish *Pelvicachromis taeniatus,* refer to [213]).

Hepper [214] demonstrated that both puppies and adults can discriminate their siblings and mother, and mothers can discriminate their offspring by olfactory cues. Adult siblings could discriminate one another only if they had co-habited. Although poorly represented in subsequent works, Hamilton and Vonk [215] specifically addressed sex differences by demonstrating that male dogs were able to discriminate kin without the prerequisite of familiarity, whereas females did not show such abilities. Although the reason for this difference remains uncertain, it should be underlined that females in the diestrus phase were tested, so the ability to discriminate kin may be activated during the estrus phase. Other studies on olfactory use have reported that male dogs tend to sniff the vaginal secretion odor more than females, whereas females investigated for longer periods of time with respect to food odor [216].

## 5. Discussion

This paper is the first comprehensive review reporting sex differences in dogs regarding personality traits, cognitive processes and perception. Although the literature is ample for some traits, thus enabling us to draw several patterns, only limited indications are present for other traits. From the data included in this review, it appears that males tend to be more aggressive and bolder than females, whereas a lower level of intraspecific sociability in males was reported. Females seem more inclined to interspecific social interactions with humans in tasks that require cooperative skills, whereas males appear more likely to interspecific social play. Studies of spatial skills underlined a higher flexibility in resorting to a particular navigation strategy in males in an outdoor environment; however, females appear to be better at spatial learning tasks in restricted areas. Lateralization studies seem to support the view that males are preferentially left-pawed and females are preferentially right-pawed; however, some studies have failed to replicate these results. Reports on visual focusing rank females as superior in focus on specific social and physical stimuli. In olfactory monitoring activity, only male dogs are able to discriminate kin. For other stimuli, the use of olfactory recording may be related to the differential relevance that olfactory signals have for males and females.

With regard to aggressiveness, it should be noted that the number of different contexts in which the dogs were tested appears to be limited. For example, it is expected that, in some circumstances (e.g., in defense of offspring), females will be more aggressive than males. Furthermore, because of the inherent differences in aggressive scores between breeds [70,217], females of some breeds might appear to be more aggressive than males of other breeds. Moreover, some studies on aggressive behaviors have been obfuscated by the methodological limitations that result from the involvement of samples that are not representative of the complete dog population (refer to [125]) or because they are based on owner surveys. Direct and indirect methods for measuring aggression have been shown to have a low reliability [53]. In contrast to the trend, a study on bite incidences toward people surprisingly indicated that female dogs were almost three times more likely to have bitten humans than male dogs, particularly in cases of small-sized species [76]. However, it should be emphasized that, in this study, a high percentage of sterilized dogs (87.5%) were used, with significantly more spayed females than castrated males. As ovariectomy results in increased aggressive behaviors in females [59,66,75,86,87,88], neutered females may have partially biased the results of Guy et al. [76]. Because of these limitations, the conclusion that male dogs are more aggressive would seem not robust. On the other side, male dogs were also reported systematically bolder than females, coherently with the behavioral syndrome with which boldness and aggressiveness positively correlate [1]. Despite the limitations in some studies, the patterns described in most of the studies are consistent with the theory of behavioral ecology, predicting that the higher level of aggression had greater positive consequences in terms of fitness for males [48]. 

Male and female dogs show different levels of sociability. Engagement in dog-human interspecific social play male dogs show more social contact than females, whereas in cooperative behavior in trying to solve a problem, the opposite trend has been found. An increased interest in intraspecific social play was identified in male primates [142,218,219]; however, it was not reported in dogs, in contrast to the sociobiological theories predicting higher social behavior in males.

Studies on spatial navigation underline that male dogs outperform females in reverting the navigation strategy in a “Do as I do” paradigm [166]; however, no sex differences were identified in reversal-learning in the T- and plus-maze paradigms [167,168]. In a T-maze paradigm, the better performance of the intact females in the learning task may be linked to the superior spatial ability of females in restricted areas reported for other mammals [158,215]. An interesting outcome in a plus-maze study is the different effect of age between sexes, with a positive correlation in females and the opposite trend in males [168]. Consistent with the reports in most mammals, females tend to disperse from their natal group less frequently than males [21]. Thus, dogs likely require more experiences across the lifespan to learn to resort to navigation strategies. In contrast, younger male dogs tend to disperse more frequently than adults [157]. Thus, it is possible that they are predisposed to use flexibly different spatial information in the early years of life to cope with unpredictable environments, in line with the major skill in the navigation strategies identified in males in other mammals [155,156] and the theoretical predictions [157,158,159]. Sexual hormones seem to affect the spatial cognition since ovariectomized females are more likely to prefer an egocentric strategy than an allocentric one [168]. An effect also underlined in rats in which a bias toward the use of egocentric rather than allocentric strategies was observed [220,221,222]. 

Most studies reported male dogs to be prevalently left-pawed, whereas females more frequently use the right paw. These outcomes are in line with the study of paw preferences in other domesticated animal species [181,182,183,185] and in captive primates [184]. The observation of no paw preference in dogs may be the result of a weak effect, making it difficult to obtain statistical significance. The right paw preference in males reported in Tan [188] may be because there were near twice as many females as males in their study and the sex as a factor was not controlled or accounted for in the analysis.

Studies on visual focusing in dogs are in line with the results from similar studies in humans and other mammals [223], which indicate that females reserve more attention for specific visual cues than males. The lower attention for single visual signals in males may be the result of greater vigilant behavior, which leads males to switch from one visual stimulus to another more often (e.g., giraffes: [202]; baboons: [201]; humans: [200]), thus not allowing them to focus their attention on a single target for a long time. Moreover, it is theorized that in the wild, male animals with a higher level of vigilance may be more attractive to choosy females because they can offer more security against predation [224,225]. There is also a male advantage from higher vigilance in preventing the behavior of sexual competitors [201,202]. Alternatively, the lower rate of visual focusing in male dogs may also be a side effect of their higher distractibility [123,126]. The higher visual focusing ability of females has direct implications for dog-human communications: owners and trainers could spend more effort on obtaining sustained visual attention from males, which is an important prerequisite to communication with dogs [226].

How do sex differences in dogs conform with the naturalistic scenario from which they originate? Along the domestication process, in which natural selection was replaced by the artificial selection, dogs seem to have maintained the sex differences in the aggressiveness-boldness syndrome described in wild animals (at least in the studied contexts). Researches in spatial cognition in restricted areas reporting better performance in females in the learning task agree with that reported for the other mammals [158,215]. Also, studies reporting male dogs to be prevalently left-pawed and females right-pawed are in line with the study of paw preferences in not domesticated species [184], but also with other domesticated animal species [181,182,183,185]. The sex differences in dog sociability seem not agree to the previsions requiring males more social, apart from the social play with humans in which male dogs seem to show a higher tendency than females. It should be emphasized that most parts of studies on sociability are centered on interspecific interaction, for which is not possible to make a comparison with wild animals. Thus, a more conservative view should be maintained for this personality trait.

In summary, the main outcome obtained by our work is that despite 30,000 years of domestication during which artificial selection was the main driver [227], dogs have largely maintained the sex differences described in wild animals. Overall, these reports suggest that sex differences in dogs are mainly rooted in their biological and evolutionary heritage. However, in contrast to the trend, these results failed to indicate an enhanced intraspecific sociability in male dogs, which may be a side effect of living in an anthropogenic niche. 

## 6. Future Directions

From our overview, it appears that studies on dog sex differences are largely biased, with some traits being largely ignored. This imposes a challenge in reaching more robust generalizations. For example, studies on olfactory skills are very limited, whereas olfactory exchanges are particularly important as communicative tools in dogs. In humans, a different use of olfactory monitoring is established, with females appearing to be more effective in the studied contexts [228], whereas in dogs, the field is completely open. This is a crucial aspect, particularly in light of the different strategies that male and female dogs adopt to achieve reproductive success.

Another field that requires exploration is the ontogenesis of personality traits. It is established that dogs appropriately socialized as puppies are less likely to exhibit aggression [57,58]; however, nothing is known about the other personality traits. The effect of the type of human-dog relationship in shaping dogs’ personalities may be very important. For example, regarding the time spent together, living conditions (home, garden), and training, the inclusion of the dogs’ sex in the variables may provide new insights and should be applied to all sex-specific variables. Some traits were more consistently investigated by many authors; however, many gaps remain to be filled, and inconsistencies in the results must be resolved.

With regard to aggressiveness, future studies should be targeted to gauge the aggressive behaviors directed toward different targets, such as familiar or unfamiliar humans, dogs, and other animals separately, because these factors seem to be uncorrelated [70,82]. Different motivations (i.e., dominance, territorial dispute, fear, and defense) should also be explored separately. The interaction of these factors should be tested in structured studies to delineate a clearer pattern. Moreover, it should be noted that in the majority of studies, the authors concentrated exclusively on physical aggression, whereas incidences not culminating in real attacks, such as threat gestures, are largely underestimated. In humans, there are indications that female criminals are more likely to commit crimes during the menstrual phase, whereas aggression is reduced around the time of ovulation [229]. Thus, there is also the need to investigate the expression of bouts of aggression in different phases of the reproductive cycle in female dogs. The effect of fluctuating hormones may well condition other personality traits, which suggests the need for additional research in this area. 

Finally, apart from the sex differences in dogs framed in a naturalist context, there are other dog-specific sex differences that require deeper exploration. One example is personality traits, such as excitability, in which females tend to be more excitable than males [123,124], distractibility, which shows an opposite trend [123,126], and cognitive abilities, in which males are reported to be smarter in a study on problem-solving [230].

## Figures and Tables

**Table 1:** List of scientific publications analyzing sex differences in dogs.

	Authors	Year	Methods	Primary Outcomes	Advantaged Sex
***Aggressiveness***	Borchelt	1983 [59]	Interview with family members	Eight major types of aggression were identified in different pure and mixed breeds: fear-elicited aggression, dominance, possessiveness, protectiveness, predation, punishment, pain and intraspecific aggression. Intraspecific and dominance aggressions as the major drivers were influenced by sex. Fear-elicited and possessive aggressions were less influenced by sex.	Males
	Hart and Hart	1985 [60]	A systematic survey of canine authorities	Males of different pure breeds showed more aggression toward other dogs.	Males
	Wright and Nesselrote	1987 [66]	Interview with family members	Males of different pure and mixed breeds showed more behavioral problems such as aggression toward dogs and humans.	Males
	Cameron	1997 [61]	Interview with the owners	Males of different pure and mixed breeds showed more dominance-associated aggression.	Males
	Guy et al.	2001 [76]	Interview with the owners	Females of different pure and mixed breeds showed more aggressive behavior toward humans.	Females
	Rooney and Bradshaw	2004 [69]	Interview with the owners and the trainers	English Springer spaniel, Labrador Retrievers, cross-breeds and Border collie males showed more aggression toward other dogs.	Males
	Pérez-Guisado et al.	2006 [62]	Experimental observation using Campbell’s test	English cocker spaniel males showed more dominance-associated aggression.	Males
	Pérez-Guisado et al.	2008a [63]	Experimental observation using Campbell’s test	Males of different pure and mixed breeds showed more dominance-associated aggression.	Males
	Pérez-Guisado et al.	2008b [64]	Interview with the owners	Males of different pure and mixed breeds showed more dominance-associated aggression.	Males
	Pérez-Guisado and Serrano	2009 [65]	Interview with the owners	Males of different pure and mixed breeds showed more dominance-associated aggression.	Males
	Foyer et al.	2013 [67]	Experimental observation	German shepherd males showed more aggressive behavior.	Males
	Lofgren et al.	2014 [68]	Interview with the owners	Labrador Retriever males showed higher owner aggression; stranger and dog-directed aggressions were not influenced by sex.	Males
	Asp et al.	2015 [70]	Interview with the owners	Males of different pure breeds showed higher stranger and dog-directed aggression.	Males
***Boldness and Courage***	Reuterwall and Ryman	1973 [122]	Interview with the trainers	German shepherd males were less impressionable by gunfire. The courage and the response to a sudden disturbance, in general, were not influenced by sex.	Males
	Goddard and Beilharz	1982 [123]	Interview with the trainers	Labrador and Golden Retriever males showed fewer fearfulness problems.	Males
	Goddard and Beilharz	1983 [124]	Interview with the trainers	Labrador and Golden Retriever males showed fewer fearfulness problems.	Males
	Goddard and Beilharz	1984 [126]	Experimental observation	Labrador Retriever, German shepherd, Boxer, Kelpie, and F1 crosses males showed less olfactory exploration associated with neophobia.	Males
	Wilsson and Sundgren	1997 [127]	Experimental observation	Labrador Retriever and German shepherd males scored higher in courage.	Males
	Svartberg	2002 [94]	Experimental observation	Belgian Tervuren and German shepherd males scored higher in boldness.	Males
	Strandberg et al.	2005 [128]	Experimental observation	Belgian Tervuren and German shepherd males scored higher in boldness.	Males
	Kubinyi et al.	2009 [130]	Interview with the owners	Males of different pure and mixed breeds scored higher in boldness.	Males
	Asp et al.	2015 [70]	Interview with the owners	Male of different pure breeds showed less dog and stranger- directed fear.	Males
***Sociability***	Lore and Eisenberg	1986 [146]	Experimental observation	Females of different pure and mixed breeds were more likely to approach and make physical contact with a human stranger. Males of different pure and mixed breeds were less likely to approach and make physical contact with a human male stranger.	Females
	Wilsson and Sundgren	1997 [127]	Experimental observation	Affability was not influenced by sex.	None
	Strandberg et al.	2005 [128]	Experimental observation	German shepherd males were more likely to social play.	Males
	Kubinyi et al.	2009 [130]	Interview with the owners	Females of different pure and mixed breeds scored higher in sociability.	Females
	Foyer et al.	2013 [67]	Experimental observation	German shepherd females scored higher in sociability.	Females
	Asp et al.	2015 [70]	Interview with the owners	Males of different pure breeds showed more human-directed play.	Males
	Persson et al.	2015 [149]	Experimental observation	Beagle females scored higher in sociability, making more physical contact with a human.	Females
	D’Aniello et al.	Pers. Comm. [150]	Experimental observation	Labrador and Golden Retriever females made more physical contact with a stranger human.	Females
***Spatial Cognition***	Fugazza et al.	2017 [165]	Experimental observation	Males of different pure and mixed breeds showed more flexibility in changing the navigation strategy from allocentric to egocentric.	Males
	Mongillo et al.	2017 [167]	Experimental observation	Females of different pure and mixed breeds learned faster and made fewer errors in learning a task in the T-maze.	Females
	Scandurra et al.	2018b [168]	Experimental observation	No effect of sex was identified on strategy preference in the plus-maze; however, an effect of gonadectomy was identified in females with a preference for the egocentric strategy in gonadectomized females. The probability of success in changing the navigation strategy increased in females and decreased in males, with increasing age.	None
***Lateralization***	Wells	2003 [189]	Experimental observation	Females of mixed breeds preferred to use the right paw, whereas males of mixed breeds were more inclined to use their left paw.	Females right pawed Males left pawed
	Quaranta et al.	2004 [190]	Experimental observation	Female of different pure and mixed breeds preferred to use the right paw, while males of different pure and mixed breeds were more inclined to adopt their left paw.	Females right pawed Males left pawed
	Branson and Rogers	2006 [193]	Experimental observation	Use of the preferred paw was not influenced by sex.	None
	Schneider et al.	2013 [194]	Experimental observation	Use of the preferred paw was not influenced by sex.	None
	Poyser et al.	2006 [195]	Experimental observation	Males of different pure and mixed breeds used the left paw more frequently; they tended to use the left paw in the first trials.	Males left pawed
	Wells et al.	2016 [191]	Experimental observation	Females of different pure and mixed breeds preferred to use the left paw, whereas males of different pure and mixed breeds were more inclined to use their right paw.	Females left pawed Males right pawed
***Visual Focusing***	Rooijakkers et al.	2009 [209]	Experimental observation	Females tended to look at the changing target longer.	None
	Müller et al.	2011 [208]	Experimental observation	Females of different pure and mixed breeds responded to a size constancy violation, looking at the changing target longer.	Females
	Nagasawa et al.	2015 [205]	Experimental observation	Females of different pure and mixed breeds showed increased gazing behavior toward the owner with intranasal oxytocin.	Females
	Kis et al.	2015 [207]	Experimental observation	Dogs of both sexes were not affected by the intranasal oxytocin.	None
	D’Aniello et al.	2016 [26]	Experimental observation	Labrador and Golden Retriever females relied more on visual signals, such as human gestural commands.	Females
	Duranton et al.	2016 [203]	Experimental observation	Females of shepherds and molossoids dogs displayed more referential gazing behavior toward the owners.	Females
	Kovács et al.	2016 [206]	Experimental observation	Females of different pure and mixed breeds increased the gazing behavior toward the owners with the intranasal oxytocin.	Females
	Mongillo et al.	2016 [204]	Experimental observation	Females of different pure and mixed breeds displayed more gazing behavior toward the owners.	Females
***Olfactory Skills***	Siniscalchi et al.	2011 [216]	Experimental observation	Males of mixed breeds tended to sniff vaginal secretion odor more frequently; females of mixed breeds investigated the food odor for a longer time.	Depending on motivation
	Hamilton and Vonk	2015 [215]	Experimental observation	Labrador, Golden Retriever and F1 crosses males were able to recognize kin.	Males
